# Calle Bengtsson in memoriam

**DOI:** 10.3109/02813432.2013.814977

**Published:** 2013-09

**Authors:** Cecilia Björkelund, Lolo Humble, Jóhann Ág. Sigurðsson

**Affiliations:** ^1^Department of Primary Health Care, University of Gothenburg, Sweden E-mail: cecilia.bjorkelund@allmed.gu.se; ^2^Department of Primary Health Care, University of Gothenburg, Sweden; ^3^Department of Family Medicine, University of Iceland, Reykjavik, Iceland

Professor, former Editor-in-Chief, Calle Bengtsson recently passed away. We, his fellow researchers, co-workers and friends, mourn and miss him. During the last 10 years of his life he was very active as professor emeritus at the University of Gothenburg and continued to participate in research in the Population Study of Women in Gothenburg, the population survey he started 40 years ago in 1968 and which is still ongoing. He participated actively in the research group meetings and contributed his extensive expertise both with ideas and also suggestions for new studies on the important facts and details from all the studies that have been generated from the Study of Women.


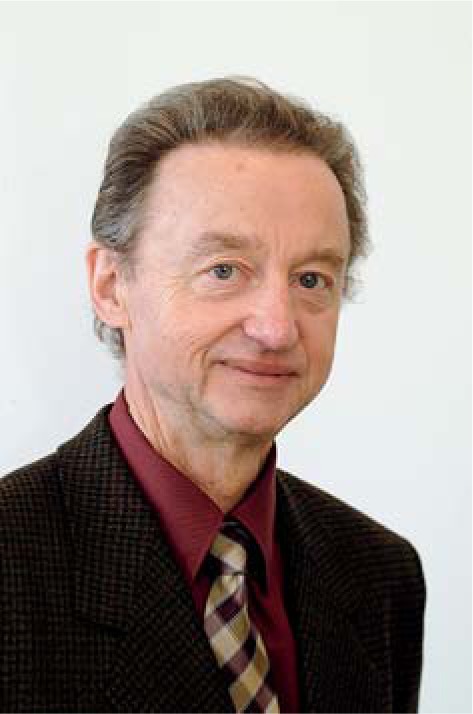


Calle Bengtsson was a farmer's son from a small rural village, where he grew up as number four of five siblings. His teacher at the elementary school strongly supported his ambition for medical training. Internal medicine was his first specialty, and it was at internal medicine at Sahlgrenska hospital in Gothenburg that he was given the task to start the Population Study of Women in Gothenburg. From 1968 Calle Bengtsson devoted much of his time as a researcher to involving 1462 women in periodic surveys to enable studies on many aspects of women's health. His research has been groundbreaking and to some extent unique – there is no population study of women in the world that has gone on for such a long time with such good participation rates and such careful follow-up.

Calle Bengtsson's broad medical expertise paired well with a great interest in people and he was a very good colleague and research group leader who constantly supported and supervised numerous graduate students and research colleagues. He was also always very keen that the women who regularly participated in the Study of Women and contributed large amounts of health data would also receive information about their health conditions and be appreciated for their participation.

Calle Bengtsson was a specialist in Family Medicine and a great representative of general practice in Sweden and Scandinavia. He was Editor-in-Chief of the Scandinavian Journal of Primary Health Care from 1996 until 2003. During this time, ownership of the journal was changed to the present state, as was the layout, and the publication rate of articles using qualitative methods increased.

His great research network also included Iceland's and Finland's medical and primary care researchers and he often visited his colleagues in Reykjavik and Finland and gave – and received – the inspiration for continued research. He was awarded a medal by the President of Iceland, and was awarded honorary doctorates by the University of Tampere.

Important achievements have been made by Calle Bengtsson's research concerning women's health. He has clearly shown that women's health is largely determined by different factors than men's health. Specifically, this applies, for example, to cardiovascular disease, in which he demonstrated that risk factors for women's cardiovascular diseases partly differ from those of men; that there is a connection between early menopause and osteoporosis; that the time after menopause is a positive time and there are not more women who have mental health problems after menopause than before; that much amalgam in teeth does not increase the risk of cancer or other diseases later in life; and that it is missing teeth rather than amalgam fillings that increase the risk of ill health.

The loss of Calle Bengtsson is great. He held meaning for us as a role model, a research colleague, and a good friend, and he will be deeply missed.

